# Conversion Strategy in Left-Sided RAS/BRAF Wild-Type Metastatic Colorectal Cancer Patients with Unresectable Liver-Limited Disease: A Multicenter Cohort Study

**DOI:** 10.3390/cancers14225513

**Published:** 2022-11-09

**Authors:** Stefano Granieri, Christian Cotsoglou, Alessandro Bonomi, Lisa Salvatore, Roberto Filippi, Olga Nigro, Fabio Gelsomino, Ina Valeria Zurlo, Ilaria Depetris, Riccardo Giampieri, Rossana Berardi, Cristina Morelli, Michele De Tursi, Michela Roberto, Elson Gjoni, Alessandro Germini, Nicola de Angelis, Riccardo Memeo, Antonio Facciorusso, Ornella Garrone, Daryl Ramai, Michele Ghidini, Alessandro Parisi

**Affiliations:** 1 General Surgery Unit, ASST-Brianza, Vimercate Hospital, Via Santi Cosma e Damiano 10, 20871 Vimercate, Italy; 2 General Surgery Residency Program, University of Milan, Via Festa del Perdono 7, 20122 Milan, Italy; 3 Facoltà di Medicina e Chirurgia, Università Cattolica del Sacro Cuore, Largo Francesco Vito 1, 00168 Rome, Italy; 4 Medical Oncology, Comprehensive Cancer Center, Fondazione Policlinico Universitario Agostino Gemelli, IRCCS, Largo Agostino Gemelli, 00168 Rome, Italy; 5 Department of Oncology, University of Turin, 10124 Torino, Italy; 6 Division of Medical Oncology, Candiolo Cancer Institute, FPO—IRCCS, 10060 Candiolo, Italy; 7 Centro Oncologico Ematologico Subalpino, Azienda Universitaria Ospedaliera Città della Salute e della Scienza di Torino, Corso Bramante 88, 10126 Turin, Italy; 8 Medical Oncology, ASST Sette Laghi, Ospedale di Circolo e Fondazione Macchi, Viale Luigi Borri 57, 21100 Varese, Italy; 9 Department of Oncology and Hematology, Division of Oncology, University Hospital of Modena, Via del Pozzo 71, 41125 Modena, Italy; 10 Medical Oncology, “Vito Fazzi” Hospital, Piazza Filippo Muratore 1, 73100 Lecce, Italy; 11 Medical Oncology, ASL TO4, Ospedale Civile di Ivrea, Piazza Credenza 2, 10015 Ivrea, Italy; 12 Clinica Oncologica e Centro Regionale di Genetica Oncologica, Università Politecnica delle Marche, AOU Ospedali Riuniti-Ancona, Via Conca 71, 60126 Ancona, Italy; 13 Medical Oncology Unit, Department of Systems Medicine, Tor Vergata University Hospital, Viale Oxford 81, 00133 Rome, Italy; 14 Department of Medical, Oral and Biotechnological Sciences and Center for Advance Studies and Technology (CAST), G. D’Annunzio University, Via dei Vestini 31, 66100 Chieti, Italy; 15 Clinical Oncology Unit, S.S. Annunziata Hospital, Via dei Vestini, 66100 Chieti, Italy; 16 Oncology Unit, Department of Clinical and Molecular Medicine, Sapienza University of Rome, Sant’Andrea Hospital, Via di Grottarossa 1035/1039, 00189 Rome, Italy; 17 Unit of Digestive Surgery, Henri Mondor Hospital, AP-HP, 1 Rue Gustave Eiffel, 94000 Créteil, France; 18 Department of Surgery, University of Paris Est Créteil (UPEC), 61 Av. du Général de Gaulle, 94000 Créteil, France; 19 Unit of Minimally Invasive and Robotic Digestive Surgery, Ospedale Regionale “F. Miulli”, 70124 Bari, Italy; 20 Gastroenterology Unit, Department of Medical Sciences, Ospedali Riuniti di Foggia, Viale Luigi Pinto 1, 71122 Foggia, Italy; 21 Operative Unit of Medical Oncology, Fondazione IRCCS Ca’ Granda Ospedale Maggiore Policlinico, Via Sforza 28, 20122 Milan, Italy; 22 Division of Gastroenterology and Hepatology, University of Utah Health, 50 Medical Dr N, Salt Lake City, UT 84132, USA; 23 Department of Life, Health and Environmental Sciences, University of L’Aquila, Piazza Santa Margherita 2, 67100 L’Aquila, Italy

**Keywords:** left colon cancer, rectal cancer, CRC, colorectal liver metastases, conversion therapy, liver resection, hepatectomy

## Abstract

**Simple Summary:**

Around 70% of patients suffering from colorectal cancer (CRC) develop liver metastases. In the present multicentric cohort study, we explored the efficacy of a conversion strategy in a selected population of 272 left-sided RAS/BRAF wild-type CRC patients with liver-limited metastatic disease. The conversion rate was 24.1%. Fifty-six patients undergoing surgical resection after induction treatment had a significant survival advantage compared to those receiving systemic treatment not leading to surgery. There was no difference in survival between ultimately resectable patients and those who had liver resection with perioperative systemic treatment. Our study confirms that in selected cases the combination of systemic treatment with surgical resection can remarkably improve survival outcomes.

**Abstract:**

Colorectal cancer (CRC) patients frequently develop liver metastases. Different treatment strategies are available according to the timing of appearance, the burden of metastatic disease, and the performance status of the patient. Systemic treatment (ST) represents the cornerstone of metastatic disease management. However, in select cases, combined ST and surgical resection can lead to remarkable survival outcomes. In the present multicentric cohort study, we explored the efficacy of a conversion strategy in a selected population of left-sided RAS/BRAF wild-type CRC patients with liver-limited metastatic disease. Methods: The primary endpoint was to compare survival outcomes of patients undergoing ST not leading to surgery, liver resection after conversion ST, and hepatic resection with perioperative ST. Furthermore, we explored survival outcomes depending on whether the case was discussed within a multidisciplinary team. Results: Between 2012 and 2020, data from 690 patients respecting the inclusion criteria were collected. Among these, 272 patients were deemed eligible for the analysis. The conversion rate was 24.1% of cases. Fifty-six (20.6%) patients undergoing surgical resection after induction treatment (i.e., ultimately resectable) had a significant survival advantage compared to those receiving systemic treatment not leading to surgery (176 pts, 64.7%) (5-year OS 60.8% and 11.7%, respectively, Log Rank test *p* < 0.001; HR = 0.273; 95% CI: 0.16–0.46; *p* < 0.001; 5-year PFS 22.2% and 6.3%, respectively, Log Rank test *p* < 0.001; HR = 0.447; 95% CI: 0.32–0.63; *p* < 0.001). There was no difference in survival between ultimately resectable patients and those who had liver resection with perioperative systemic treatment (potentially resectable—40 pts) (5-year OS 71.1%, Log Rank test *p* = 0.311. HR = 0.671; 95% CI: 0.31–1.46; *p* = 0.314; 5-year PFS 25.7%, Log Rank test *p* = 0.305. HR = 0.782; 95% CI: 0.49–1.25; *p* = 0.306). Conclusions: In our selected population of left-sided RAS/BRAF wild-type colorectal cancer patients with liver-limited disease, a conversion strategy was confirmed to provide a survival benefit. Patients not deemed surgical candidates at the time of diagnosis and patients judged resectable with perioperative systemic treatment have similar survival outcomes.

## 1. Introduction

Fourteen to eighteen percent of patients suffering from colorectal cancer (CRC) present metastases at the time of diagnosis [1]. Approximately 70% of CRC patients experience metastatic disease to the liver [1,2]. According to major guidelines, the preferred option as first-line treatment for patients with unresectable left-sided *RAS* and *BRAF* wild-type metastatic colorectal cancer (mCRC) is represented by a cytotoxic fluoropyrimidine-based doublet (FOLFOX or FOLFIRI) in association with an Anti-epidermal growth factor receptor monoclonal antibody (i.e., EGFRi, cetuximab, or panitumumab) [3,4]. In this regard, a landmark retrospective pooled analysis of six randomized phase III trials showed a significant survival benefit for doublet plus EGFRi compared to doublet plus bevacizumab in *RAS* wild-type tumors originating from the left side of the colon, and this trend has been confirmed in real-life settings [5,6]. Although systemic treatment remains the cornerstone of metastatic disease management, with different emerging strategies after induction treatment in the never-resectable disease [7], surgical resection has progressively gained interest in the scientific community.

Treatment options include simultaneous resection of the primary tumor and liver in light of synchronous disease or a staged approach for both synchronous and metachronous diseases [8]. The latter encompasses upfront resection of the primary tumor or liver resection followed by adjuvant systemic treatment (ST) before bowel resection or perioperative ST with liver resection followed by bowel resection. Unfortunately, only 10–20% of patients developing colorectal liver metastases (CRLM) are deemed eligible for liver resection, mainly due to heavy disease burden; however, promising results have been reported in terms of conversion rate after the administration of highly effective chemotherapy (CT) regimens with or without targeted agents [9,10,11,12,13,14]. 

Over the years, multiple guidelines and consensus have stratified CRLM patients based on clinical and biological characteristics of the tumor, technical resectability, and spread of the disease. While patients suffering from metastatic liver disease with good prognostic features (single and small lesions) are considered eligible for upfront surgical resection, patients with biologically more aggressive disease and/or presenting metastases adjacent to major vascular/biliary structures (borderline/potentially resectable) may benefit from perioperative ST. In case the spread of the disease hinders the possibility of surgical resection with curative intent, doublet or triplet ST regimens should be adopted to induce tumor shrinkage leading to surgical resection (initially unresectable/ultimately resectable patients) [15,16,17,18].

The aim of the present study is to retrospectively assess the efficacy of conversion therapy to prolong survival in a molecularly selected population of left-sided RAS/BRAF wild-type mCRC patients with liver-limited disease, according to the abovementioned classification and treated with doublet chemotherapy plus EGFRi.

## 2. Methods

### 2.1. Study Population

In the present multi-institutional study, data were retrospectively collected from patients diagnosed with liver metastases due to RAS/BRAF wild-type left-sided colon or rectal cancer and consecutively treated from March 2012 to October 2020. 

### 2.2. Study Design

Demographics, clinicopathology, tumor biology, disease burden, treatment strategy, neoadjuvant treatments, surgical details, postoperative treatments, disease relapse, disease-related death, and follow-up information were gathered to build the dataset. 

Baseline resectability of CRC liver metastases was assessed by surgeons, radiologists, and oncologists of each center, within or outside a defined multidisciplinary team (MDT), according to their local clinical practice. In case of baseline technical resectability (i.e., major hepatectomy, lobectomy, or other demolitive surgery not required), the decision to administer a preoperative treatment was related to the presence of biologically challenging criteria [19].

For convenience, the classification provided by Bittoni et al. and the Fong criteria were adopted [18,20]. Based on disease burden, patients were a posteriori classified as potentially resectable or unresectable. 

Patients were defined as “potentially resectable” either when presenting biological challenges according to FONG criteria (multiple metastases, size > 5 cm, positive lymph nodes at primary tumor, synchronous metastases or DFS < 12 months, CEA > 100 ng/mL, as assessed at baseline before beginning the systemic treatment) or technical challenges (tumor close to hepatic veins or portal branches, major hepatectomy required). These patients were considered eligible for perioperative chemotherapy and subsequent surgical resection.

On the other hand, patients judged as unresectable have been further classified as ultimately resectable (>70–80% of liver involvement, <25% remnant after resection, and six segments involved) or never resectable (unresectable extrahepatic disease). The former was deemed candidates for a conversion strategy in the presence of sufficient response; the latter was offered palliative chemotherapy.

CRLMs were defined as synchronous or metachronous according to Adam et al. [21]. Major hepatectomy was defined by the resection of four or more segments according to Reddy [22]. Progression-free survival (PFS) was defined as the duration from the beginning of the first-line treatment to disease progression, to death resulting from any cause, or to the last contact. Overall survival (OS) was defined as the duration between the beginning of first-line treatment to death resulting from any cause or to the last contact. For PFS as well as OS, patients without events were considered censored at the time of their last follow-up.

The primary endpoint was to compare survival outcomes (OS and PFS) of patients undergoing systemic treatment not leading to liver resection and that of surgically resected patients. Furthermore, we explored survival outcomes of ultimately resectable and potentially resectable patients.

As secondary endpoints, we investigated whether survival outcomes were different between patients whose strategy was discussed or not in MDT. As an exploratory outcome, we investigated eventual survival differences among unresected, ultimately resectable, and potentially resectable patients by stratifying these subgroups for induction ST regimen. 

Patients with missing survival and recurrence information were excluded from data collection.

### 2.3. Statistical Analysis

Sample distribution was evaluated with Kolmogorov–Smirnov and Shapiro–Wilk tests. Continuous variables were compared by Kruskall–Wallis or ANOVA when appropriate, while categorical variables were analyzed using Pearson’s chi-squared test. Tests were adjusted for all pairwise comparisons using the Bonferroni correction.

Survival curves were obtained through the Kaplan–Meier method, and log-rank test was used to evaluate differences in cumulative survival among groups. A logistic regression model was built to detect independent predictors of outcome and to estimate the adjusted odds ratio (OR) and 95% confidence interval (95% CI). Only significant (*p* < 0.1) variables in the univariate analysis were included in the multivariate model. The median period of follow-up was calculated through the reverse Kaplan–Meier method. *p* values below 0.05 were considered statistically significant. Data were recorded in a computerized spreadsheet (Microsoft Excel 2016; Microsoft Corporation, Redmond, WA, USA) and analyzed with statistical software (IBM Corp., released 2021, IBM SPSS Statistics for Windows, Version 27.0; Armonk, NY, USA, IBM Corp.).

## 3. Results

### 3.1. Descriptive Statistics of the Sample

From March 2012 to October 2018, data have been recorded from 690 patients diagnosed with left-sided RAS/BRAF wild-type mCRC and treated with EGFRi-based first-line doublet chemotherapy. Three-hundred ninety-eight patients did not meet the inclusion criteria, and two-hundred ninety-two patients had liver-limited disease. Among these, 232 patients were judged as unresectable at diagnosis, but eventually, 56 of them had radical liver resection. Therefore, the proportion of patients undergoing chemotherapy leading to surgery with a curative intent (conversion rate) was 24.1%.

**Figure 1:**
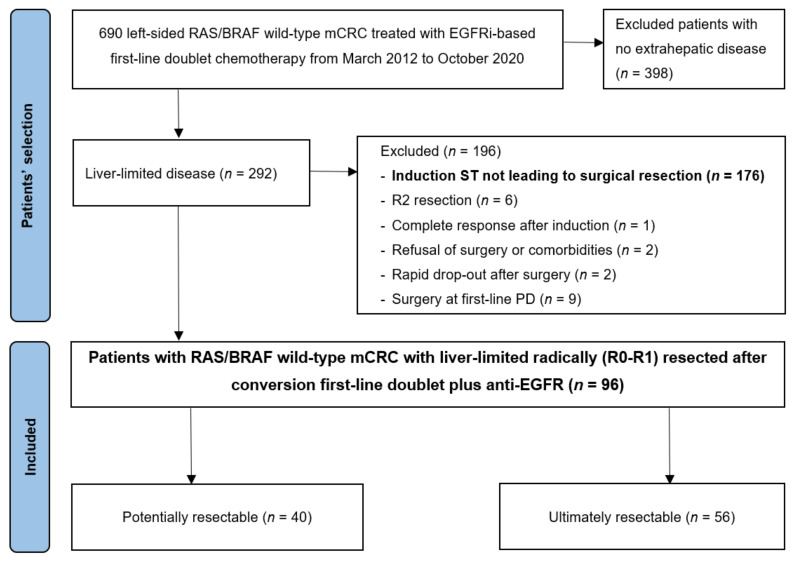
Flow diagram with patients’ selection and disposition. Three out of six R2 patients were resected with a palliative intent. ST: systemic treatment.

Further information is reported in the flow diagram depicting patients’ selection (Figure 1).

Among patients with liver-limited metastatic disease, only 96 (32.5%) underwent liver resection. Within this subgroup, 40 (41.7%) patients were deemed potentially resectable at diagnosis, whereas 56 (58.3%) patients were classified as ultimately resectable.

All patients initially identified as potentially resectable were eventually resected.

Compared to unresected patients, patients within the ultimately resectable cohort were more likely to be male (*p* = 0.03); ultimately and potentially resectable patients had a significantly greater proportion of primary tumor resected (*p* < 0.001); unresected patients had greater nodal involvement (*p* = 0.002) and a higher burden of liver disease (proportion of >10 metastases at diagnosis—*p* < 0.001); and moreover, unresected patients had greater frequency of baseline CEA > 100 ng/mL compared to potentially resectable ones (*p* = 0.016). Further characteristics of the sample are reported in Table 1.

### 3.2. Primary Endpoint

The median period of follow-up was 35.4 months (95% CI: 30.6–40.2) in the overall population, 33.8 months (95% CI: 26.1–41.4) for patients undergoing ST not leading to surgery, 34.5 months (95% CI: 27.1–41.9) for the ultimately resectable group, and 43.9 months (95% CI: 35.2–52.6) for the potentially resectable group.

Patients undergoing liver resection after induction ST had a remarkable survival advantage compared to those receiving ST not leading to surgery (Log Rank test *p* < 0.001). The 5-year OS was 60.8% and 11.7%, respectively, (HR = 0.273; 95% CI: 0.16–0.46; *p* < 0.001). The former group had a median OS that was more than double compared to the latter (79.4 months, 95% CI: *not reached* vs. 28.0 months, 95% CI: 22.14–33.93). Potentially resectable patients had 71.1% of 5-year OS and demonstrated a significant survival advantage compared to patients not leading to resection (Log Rank test *p* < 0.001. HR = 0.443; 95% CI: 0.32–0.62; *p* < 0.001). The median OS was 94.5 months (95% CI: 49.95–139.19). Compared to those belonging to the ultimately resectable group, no survival differences were observed (Log Rank test *p* = 0.311. HR = 0.671; 95% CI: 0.31–1.46; *p* = 0.314).

**Figure 2:**
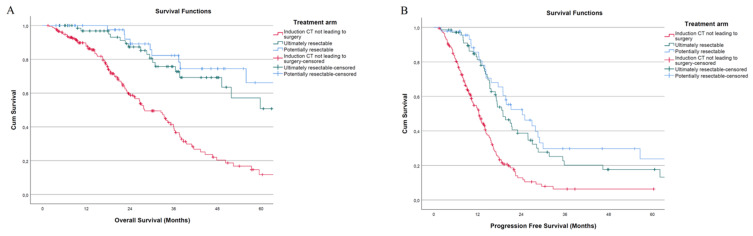
Kaplan–Meier curves of different treatment arms: (**A**) 5-year OS; (**B**) 5-year PFS.

Similarly, ultimately resectable patients had a significantly higher PFS compared to those receiving ST not leading to surgery (Log Rank test *p* < 0.001). The 5-year PFS was 22.2% and 6.3%, respectively (HR = 0.447; 95% CI: 0.32–0.63; *p* < 0.001). Ultimately resectable patients had a median PFS of 19 months (95% CI: 14.85–23.18), whereas the median was 12.4 months (95% CI: 10.75–14.02) for unresected patients. The 5-year PFS for potentially resectable patients was 25.7%, with a median PFS time of 24.3 months (95% CI: 15.27–33.35). No difference in PFS was observed compared to patients belonging to the ultimately resectable group (Log Rank test *p* = 0.305. HR = 0.782; 95% CI: 0.49–1.25; *p* = 0.306), although it was significantly higher compared to unresected patients (Log Rank test *p* < 0.001. HR = 0.605; 95% CI: 0.49–0.75; *p* < 0.001). Kaplan–Meier curves depicting 5-year OS and PFS are reported in Figure 2. Survival outcomes of R2 patients are reported in the Supplementary Materials (Figures S1–S4).

### 3.3. Secondary Endpoints

**Figure 3:**
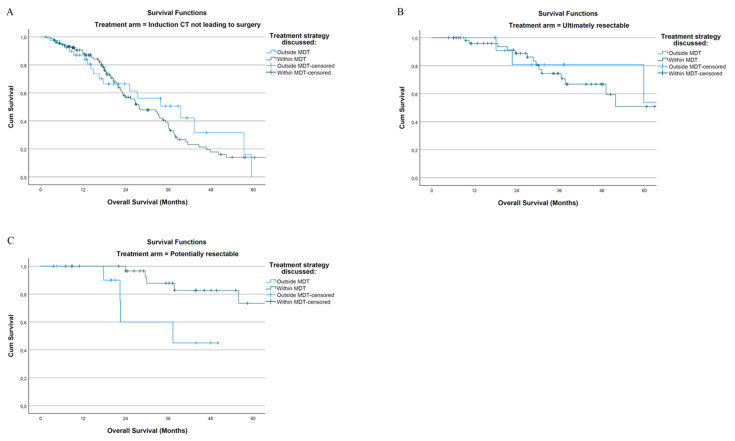
Kaplan–Meier curves of patients discussed or not in MDT—5-year OS of (**A**) unresected patients; (**B**) ultimately resectable patients; (**C**) potentially resectable patients.

Despite the improved 5-year OS for patients whose treatment strategy was discussed in MDT compared to their counterparts not discussed in MDT (14% vs. 0% for unresected patients; 61.9% vs. 60% for ultimately resectable; 75.7% vs. 60%—at 48 months—for potentially resectable), pairwise Kaplan–Meier analysis demonstrated significant differences only in the potentially resectable group (Log Rank test *p* = 0.641 for unresected patients; = 0.743 for ultimately resectable; = 0.012 for potentially resectable) (Figure 3A–C).

There was no difference in 5-year PFS highlighted among the three groups (Log Rank test *p* = 0.345 for unresected patients; =0.936 for ultimately resectable; =0.243 for potentially resectable) (Kaplan–Meier curves are reported in the Supplementary Materials—Figure S5).

Compared to unresected patients, a survival advantage (for both OS and PFS) was noticed for patients belonging to potentially and ultimately resectable groups and receiving either FOLFIRI- or FOLFOX-based I line ST regimens. However, patients receiving FOLFIRI-based regimens had a significant survival benefit compared to their counterparts receiving FOLFOX (Log-rank test *p* = 0.039). Five-year OS and PFS Kaplan–Meier curves are reported in the Supplementary Materials (Figures S6 and S7).

## 4. Discussion

In the present multicenter cohort study, 292 patients with RAS/BRAF wild-type liver-limited metastatic disease starting from left-sided CRC were analyzed. Fifty-six patients initially judged as non-surgical candidates eventually underwent hepatic resection with a conversion rate of 24.14%. A survival advantage was demonstrated for these patients over those receiving ST not leading to surgery. 

Although our findings may not appear original, the present study may represent a valuable confirmation of current international guidelines. Multiple high-volume RCTs enrolled mCRC patients treated with doublets or triplets regimens, with or without surgical resection. Nevertheless, in most cases, these studies included both patients suffering from left- and right-sided colon cancer, regardless of RAS/BRAF mutational status. In our study, only a highly selected cohort of patients with left-sided and RAS/BRAF wild-type tumors was included, with survival outcomes way above the average reported in other trials.

In addition, there was no difference demonstrated between patients receiving perioperative ST (potentially resectable) and ultimately resectable.

In this regard, it is worth noting that patients included in our study were all potentially resectable or non-resectable at diagnosis. This means that upfront surgery was not indicated, and patients were offered first-line medical treatment including EGFRi. Indeed, as demonstrated by the results of the New EPOC trial, in mCRC patients with resectable liver disease, the standard of care should be chemotherapy without monoclonal antibodies [23]. In contrast to our study, the New EPOC trial included patients that had a suboptimally resectable disease at diagnosis. Moreover, the New EPOC trial included only KRAS wild-type mCRC patients; meanwhile, our study included patients with both RAS and BRAF wild-type status.

In past years, conversion chemotherapy has led to surgical resection in up to 14% of cases [24,25,26]. In the PRIME trial, the complete resection rate for patients with KRAS WT mCRC was 10% (31 of 325 patients) in the panitumumab–FOLFOX4 arm [24]. In the PEAK trial, patients with RAS WT tumors receiving panitumumab + mFOLFOX6 had longer PFS (12.8 vs. 10.1 months, *p* = 0.029) and duration of response (11.4 vs. 9.0 months, *p* = 0.011) compared to those receiving bevacizumab + mFOLFOX6. The resection rates were similar between treatment arms (14 vs. 11%) [27].

In the last three years, several trials have demonstrated the possibility of increasing the proportion of patients converting to resectability [28,29,30,31,32,33,34]. 

Although many studies have tried to detect differences in survival related to the first-line chemotherapeutic backbone, the definition of the optimal ST regimen is still a matter of debate. Our results suggest that resected patients have a survival benefit compared to their unresected counterparts regardless of the subtype of doublet regimen. Nevertheless, looking more thoroughly at the ultimately resectable subgroup, patients receiving EGFRi/FOLFIRI-based regimens had a significant survival advantage compared to those receiving EGFRi/FOLFOX.

Compared to recent RCTs based on doublets with EGFRi/anti-VEGF, our results appear to be consistent. In the BECOME trial, the rate of conversion to surgery of patients treated with mFOLFOX6 plus Bevacizumab reached 23.1% [29]. In this trial, the OS and PFS of patients undergoing ST leading to surgery were 59.2% and 22.2%, respectively. In our study, the 5-year OS was 60.8% and the 5-year PFS was 22.2%. The median OS and PFS of these patients were 37.8 months and 7.8 months, which is remarkably lower compared to the 79.4 months OS and 21.5 months PFS of our study population. Nevertheless, it should be noted that the BECOME trial enrolled not only left-sided CRLM patients but, more importantly, RAS-mutated patients.

Looking at the results of a recent retrospective study conducted at the Karolinska University Hospital [35], 31 patients over 100 receiving conversion ST became eligible for liver resection. Despite a higher conversion rate, 5-year OS and PFS, and median OS and PFS of the three groups (unresected, resected after conversion ST, and resected after neoadjuvant ST) were inferior. For completeness, it is worth mentioning that the study by Villard et al. included both right- and left-sided primary tumors and RAS/BRAF-mutated patients. 

While research on optimal conversion ST is still on-going, different RCTs explored the impact of triplet regimens on survival outcomes and tumor response. Highly effective regimens, mainly based on FOLFOXIRI in association with Bevacizumab, led patients (initially unresectable) to being eligible for hepatectomy in up to 49% of cases, with a median PFS up to almost 32 months [31]. Notwithstanding, the efficacy of such intensive regimens is paid in terms of adverse events, especially ST-induced liver injury (steatohepatitis and sinusoidal damage), which may conflict with extensive hepatic resection. Therefore, as recommended by the most recent international guidelines [36], the main aim of any active metastatic systemic regimen should be obtaining sufficient downsizing of liver disease to allow for prompt surgical resection.

According to these principles, different studies have tried to explore the impact of several factors at the baseline evaluation that can stratify response to chemotherapy in terms of survival outcomes. In this scenario, Fong criteria were among the first to be proposed to predict cancer recurrence after liver resection, the encompassed nodal status, the number of CRLMs, the size of the largest metastasis, CEA level, and disease-free interval < 12 months after diagnosis of primary colorectal cancer [20]. In recent years, the GAME score has been presented and includes several other preoperative parameters (such as RAS mutational status and the presence of extrahepatic disease). The score outperformed Fong criteria in predicting overall survival of CRLM patients [37]. Finally, in 2020, the Comprehensive Evaluation of Relapse Risk (CERR) score was developed and validated. It evaluates the presence of KRAS, NRAS, and BRAF mutations, nodal status, CEA/CA19-9 levels, and the modified Tumor Burden Score to predict cancer recurrence. To this end, the CERR score was significantly superior to Fong and GAME scores in predicting overall survival [38].

In this setting, it would be compelling to explore the role of the parameters constituting the aforementioned tools to predict the conversion to resectability. We can argue that patients defined as low risk would be the ones who would best respond to induction ST. Therefore, in such cases, active doublet + EGFRi regimens with a lower toxicity profile would be preferable over more effective, but more toxic schemes, to achieve conversion to resectability. Indeed, patients with more aggressive tumor burden and biology would require more aggressive chemotherapy given the lower rates of conversion to resectability [24,25,39].

The enrollment of highly selected patients may have influenced our results. In our series, only patients suffering from left-sided CRLMs were included. The location of the primary tumor is currently a well-defined prognostic and predictive factor of response to EGFRi [5,40,41,42]. A recent meta-analysis highlighted how left-sided primaries achieve better outcomes (OS, PFS, and objective response rate) compared to right-sided primaries when ST plus EGFRi is administered. 

In addition, patients enrolled in the present study were all RAS/BRAF wild type, which is another well-recognized positive prognostic factor. Furthermore, in our series, patients receiving induction ST not leading to surgery had significantly greater nodal involvement, hepatic disease load (in terms of number of liver metastases), and CEA levels at baseline which are all well-established predictors of worse outcomes.

The importance of multidisciplinary teams to improve survival outcomes of stage IV colorectal cancer patients is currently well-defined, and the involvement of experienced hepatobiliary surgeons has demonstrated a trend leading towards higher resection rates and even more improved survival rates [3,36,43,44]. Surprisingly, in our series, no differences in OS and PFS were found between patients discussed within and outside MDT for each subgroup of patients. However, this result should not undermine the consolidated and remarkable effectiveness of multi-disciplinary teams; indeed, treatment strategy was decided outside MDT in only about 20% of cases, mostly belonging to unresected patients group. Thus, such a limited proportion may have influenced the lack of significant differences between the two groups.

Our study has limitations worth mentioning; first, its retrospective nature. The main issue is represented by the lack of a standardized definition of resectability. In 2006, Charnsangavej proposed three criteria to define resectability: preservation of at least two contiguous hepatic segments, adequate blood flow and biliary drainage, and >20% remnant liver of total liver volume. In our series, although in 78.1% of cases, the treatment strategy was discussed at MDT, it was not possible to determine whether the above-mentioned criteria were observed or not, or if other criteria were adopted to define resectability. Nevertheless, in all participant centers, a specialized hepato-biliary surgery unit was present; therefore, we can hypothesize that patients were evaluated by experienced liver surgeons, regardless of whether the assessment was provided within or outside MDT. According to Wei, the use of an MDT represents an important aspect of offering CRLM patients adequate and tailored treatment [45].

Another pitfall related to the retrospective nature of the study regards the subset of patients undergoing bowel resection first. To this end, it was not possible to define the reason for such a treatment approach (elective proper colectomy first or urgent/emergent colectomy for occlusion, perforation, and bleeding). 

A further drawback of our study may be represented by the adoption of Fong criteria, which although valuable and widely used over the last two decades, have been developed more than twenty years ago. Unfortunately, we were not able to compute GAME and CERR scores due to the lack of several data parameters that were not collected because the aforementioned tools were first published only after the end of data collection.

### Future Perspectives

Given the very poor survival outcomes of systemic therapy alone, growing evidence towards liver transplantation for even more strictly selected patients with nonresectable CRLM is being reported. Liver transplantation might be an additional therapeutic option for patients responding to induction ST but not achieving eligibility for resection, with a great advantage in terms of survival outcome compared to chemotherapy alone. Patient selection based on a combination of clinical and patho-radiological criteria (i.e., metabolic tumor volume and total lesion glycolysis on PET), molecular prognostic markers, and dynamic evaluation of biological behavior (assessment of response to bridging therapy during an extended period of observation time) guarantees 5-year OS ranging from 50% to 100% for unresectable liver disease [46,47,48]. Notably, the inclusion criteria for liver transplantation in ongoing trials enrolling unresectable CRLM are, like those described in our study, to identify patients that are eligible for successful conversion therapy.

However, there is little evidence to support liver transplantation over surgical resection for patients with resectable colorectal liver metastases after conversion therapy: if induction ST succeeds in making the patient resectable, surgical resection should be the preferred choice (even with sequential resection techniques such as two-stage hepatectomy and associating liver partition and portal vein ligation for staged hepatectomy) [49]. This is also true considering the shortage of donors and the unsolved problem of organ allocation.

## 5. Conclusions

Left-sided RAS/BRAF wild-type metastatic colorectal cancer patients with unresectable liver-limited disease at diagnosis may represent a subset of ideal candidates for tailored conversion strategies with the best available benefit–risk ratio, especially those with favorable biological behavior. Future prospective trials elucidating the role of different biological prognostic factors are needed to improve risk stratification. 

## Figures and Tables

**Table 1:** Clinicopathological, preoperative, and surgical characteristics of CRLM patients undergoing liver resection.

Variable	Induction ST not Leading to Surgery (*n* = 176) N (%)	Ultimately Resectable (*n* = 56) N (%)	Potentially Resectable (*n* = 40) N (%)	Total (*n* = 272) N (%)	*p*
Age (median/IQR)	65 (57.3–72.7)	62 (52.8–70.7)	62.5 (57.2–69.7)	64 (56–71)	0.34
Male gender	110 (62.5)	46 (81.1) †	29 (72.5)	185 (68.0)	*0.03*
Site of primary tumor (rectum)	54 (30.7)	21 (38.9)	17 (44.7)	92 (34.3)	0.186
Histology (mucinous)	31 (18.5)	3 (5.7)	7 (18.4)	41 (15.8)	0.075
Grading					0.63
G1	7 (5.0)	1 (2.1)	1 (3.2)	9 (4.1)	
G2	96 (68.1)	38 (79.2)	21 (67.7)	155 (70.5)	
G3	38 (27.0)	9 (18.8)	9 (29.0)	56 (25.5)	
Primary tumor resected	115 (65.7)	52 (96.3) †	36 (94.7) ‡	203 (76.0)	**<0.001**
N+	65 (36.9) ^⁑	10 (18.5)	5 (13.2)	80 (29.9)	**0.002**
N° of liver metastases at diagnosis					**<0.001**
1	9 (5.1)	5 (8.9)	10 (25.0) ‡	24 (8.8)	
2–5	57 (32.4)	28 (50.0)	22 (55.5) ‡	107 (39.3)	
6–10	32 (18.2)	9 (16.1)	3 (7.5)	44 (16.2)	
>10	59 (33.5) ⁑	13 (23.2)	4 (10)	76 (27.9)	
Synchronous disease	150 (85.2)	45 (80.4)	28 (70.0)	223 (82.0)	0.073
Treatment approach discussed at MDT	136 (77.3)	43 (76.8)	32 (80)	211 (77.6)	0.921
ECOG 0	103 (58.5)	40 (71.4)	31 (32.3)	171 (63.8)	0.089
CEA at baseline (>100 ng/mL)	60 (37.5) ⁑	15 (30)	4 (12.1)	79 (32.5)	**0.016**
HER-2 status					0.069
Not amplified	25 (14.2)	15 (27.8)	3 (7.8)	43 (16)	
Amplified	3 (1.7)	1 (1.9)	0	4 (1.5)	
Not evaluated	148 (84.1)	38 (70.4)	35 (92.1)	221 (82.5)	
N° of criteria for biologically challenging disease (median/IQR)	3 (2–3)	2 (2–3)	2 (2–3)	2 (2–3)	0.075
Major liver resection	**-**	30 (56.6)	13 (34.2)	43 (44.8)	0.038
R0 resection	**-**	39 (78)	34 (89.5)	73 (76.0)	0.293
ST regimen					0.49
FOLFIRI + Cetuximab	71 (40.3)	21 (37.5)	20 (51.3)	112 (41.5)	
FOLFOX + Cetuximab	23 (13.1)	5 (8.9)	2 (5.1)	30 (11.1)	
FOLFIRI + Panitumumab	12 (6.8)	3 (5.4)	1 (2.6)	16 (5.9)	
FOLFOX + Panitumumab	70 (39.8)	27 (48.2)	15 (38.5)	112 (41.5)	
Number of ST cycles administered before surgical resection (median/IQR)	5 (1.5–10.5)	8 (6–12)	6 (0–8)	7 (5–10)	**0.002** *
3 months PFS	167 (94.9)	56 (100)	40 (100)	263 (96.7)	0.083
6 months PFS	138 (78.4)	54 (98.2) †	37 (97.4) ‡	229 (85.1)	**<0.001**
10 months PFS	95 (54.0)	46 (83.6) †	34 (89.5) ‡	175 (65.1)	**<0.001**

PFS: Progression Free Survival; ECOG: Eastern Cooperative Oncology Group; ST: Systemic Treatment; SD: Standard Deviation; IQR: Interquartile range; * pairwise comparison favoring ultimately resectable over potentially resectable; † pairwise comparison favoring ultimately resectable over induction CT not leading to surgery; ‡ pairwise comparison favoring potentially resectable over induction CT not leading to surgery; ^ pairwise comparison favoring induction CT not leading to surgery over ultimately resectable; ⁑ pairwise comparison favoring induction CT not leading to surgery over potentially resectable. Significant *p*-values are evidenced in bold.

## Data Availability

The datasets used during the present study are available from the corresponding authors upon reasonable request.

## Supplementary Materials

The following supporting information can be downloaded at https://www.mdpi.com/article/10.3390/cancers14225513/s1; Kaplan-Meier curves for OS including R2 resections with curative intent (page 2 supplementary file); Kaplan-Meier curves for PFS including R2 resections with curative intent (page 3 supplementary file); Kaplan-Meier curves for OS including all R2 resections (page 4 supplementary file); Kaplan-Meier curves for PFS including all R2 resections (page 5 supplementary file); Kaplan–Meier curves for PFS of patients discussed within and outside MDT (page 6 supplementary file); Kaplan–Meier curves for OS of patients receiving different I line ST regimens (page 8 supplementary file); and Kaplan–Meier curves for PFS of patients receiving different I line ST regimens (page 10 supplementary file).
