# Supplementary material for: Conversion Strategy in Left-Sided RAS/BRAF Wild-Type Metastatic Colorectal Cancer Patients with Unresectable Liver-Limited Disease: A Multicenter Cohort Study
Source: Cancers (Basel). 2022 Nov 9;14(22):5513. doi: 10.3390/cancers14225513 (PMC9688791; doi:10.3390/cancers14225513)
Supplement: Supplementary file 1 [file cancers-14-05513-s001.zip › cancers-1950287-supplementary.pdf]

Conversion strategy in left-sided RAS/BRAF wild type metastatic colorectal cancer patients with liver-limited disease: a multicenter cohort study

Supplementary materials

INDEX

1) Figure S1: Kaplan-Meier curves for OS including R2 resections with curative intent	2
2) Figure S2: Kaplan-Meier curves for PFS including R2 resections with curative intent	3
3) Figure S3: Kaplan-Meier curves for OS including all R2 resections	4
4) Figure S4: Kaplan-Meier curves for PFS including all R2 resections	5
5) Figure S5: Kaplan-Meier curves for PFS of patients discussed within and outside MDT	6
6) Figure S6: Kaplan-Meier curves for OS of patients receiving different I line ST regimens	8
7) Figure S7: Kaplan-Meier curves for PFS of patients receiving different I line ST regimens	10

1) Figure S1: Kaplan-Meier curves for OS including R2 resections with curative intent

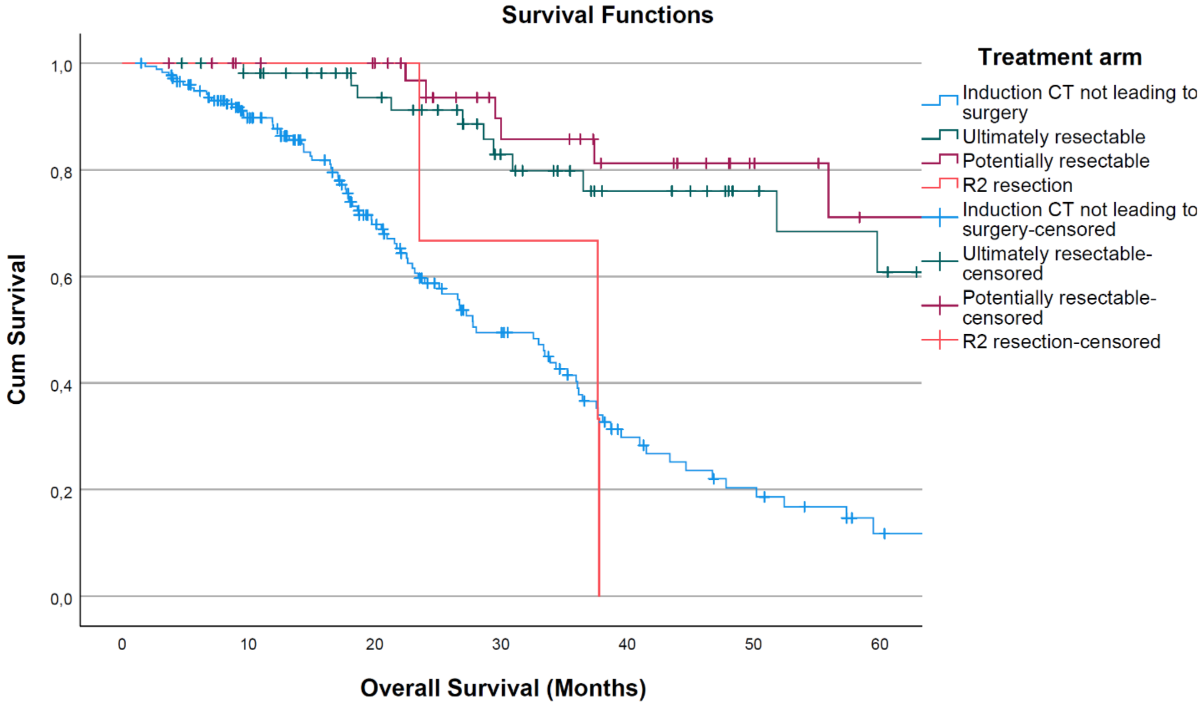

Means and Medians for Survival Time

Treatment arm	Mean[a] Estimate	Mean Std. Error	Mean 95% Confidence Interval Lower Bound	Mean 95% Confidence Interval Upper Bound	Median Estimate	Median Std. Error	Median 95% Confidence Interval Lower Bound	Median 95% Confidence Interval Upper Bound
Induction CT not leading to surgery	33,078	1,918	29,318	36,838	28,039	3,007	22,145	33,933
Ultimately resectable	62,974	4,336	54,476	71,472	79,379	,000	.	.
Potentially resectable	83,119	8,212	67,024	99,213	.	.	.	.
R2 resection	32,985	4,728	23,718	42,251	37,647	11,527	15,054	60,240
Overall	47,831	3,515	40,942	54,720	37,778	2,638	32,606	42,949

a. Estimation is limited to the largest survival time if it is censored.

Pairwise Comparisons

	Treatment arm	Induction CT not leading to surgery Chi-Square	Induction CT not leading to surgery Sig.	Ultimately resectable Chi-Square	Ultimately resectable Sig.	Potentially resectable Chi-Square	Potentially resectable Sig.	Ultimately resectable - R2 resection Chi-Square	Ultimately resectable - R2 resection Sig.
Log Rank (Mantel-Cox)	Induction CT not leading to surgery			29,582	<,001	29,187	<,001	,043	,836
	Ultimately resectable	29,582	<,001			,588	,443	6,314	,012
	Potentially resectable	29,187	<,001	,588	,443			9,352	,002
	R2 resection	,043	,836	6,314	,012	9,352	,002		

2) Figure S2: Kaplan-Meier curves for PFS including R2 resections with curative intent

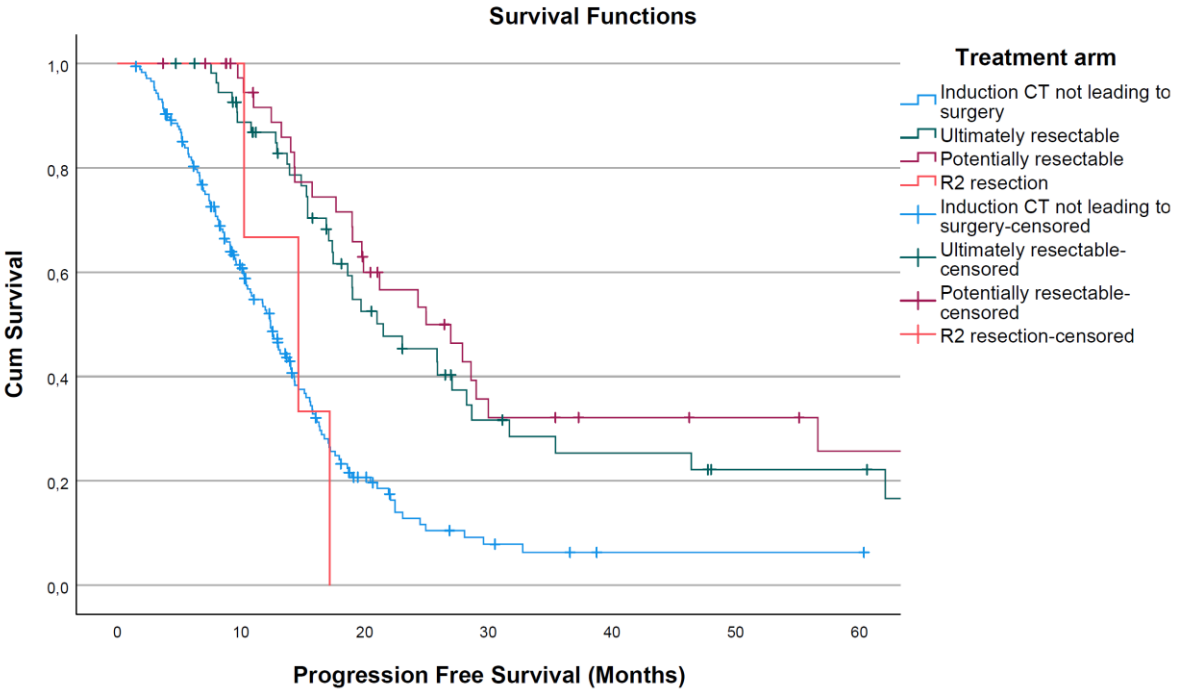

Means and Medians for Survival Time

Treatment arm	Mean[a] Estimate	Mean Std. Error	Mean 95% Confidence Interval Lower Bound	Mean 95% Confidence Interval Upper Bound	Median Estimate	Median Std. Error	Median 95% Confidence Interval Lower Bound	Median 95% Confidence Interval Upper Bound
Induction CT not leading to surgery	15,483	1,250	13,034	17,932	12,386	,832	10,754	14,017
Ultimately resectable	31,831	3,705	24,570	39,093	21,503	3,548	14,549	28,458
Potentially resectable	42,152	6,813	28,798	55,507	24,967	4,221	16,695	33,240
R2 resection	14,031	2,023	10,065	17,996	14,641	3,576	7,633	21,649
Overall	25,725	2,288	21,240	30,210	15,392	,732	13,958	16,826

a. Estimation is limited to the largest survival time if it is censored.

Pairwise Comparisons

	Treatment arm	Induction CT not leading to surgery Chi-Square	Induction CT not leading to surgery Sig.	Ultimately resectable Chi-Square	Ultimately resectable Sig.	Potentially resectable Chi-Square	Potentially resectable Sig.	Ultimately resectable - R2 resection Chi-Square	Ultimately resectable - R2 resection Sig.
Log Rank (Mantel-Cox)	Induction CT not leading to surgery			25,718	<,001	25,944	<,001	,009	,923
	Ultimately resectable	25,718	<,001			,645	,422	5,445	,020
	Potentially resectable	25,944	<,001	,645	,422			7,240	,007
	R2 resection	,009	,923	5,445	,020	7,240	,007		

3) Figure S3: Kaplan-Meier curves for OS including all R2 resections

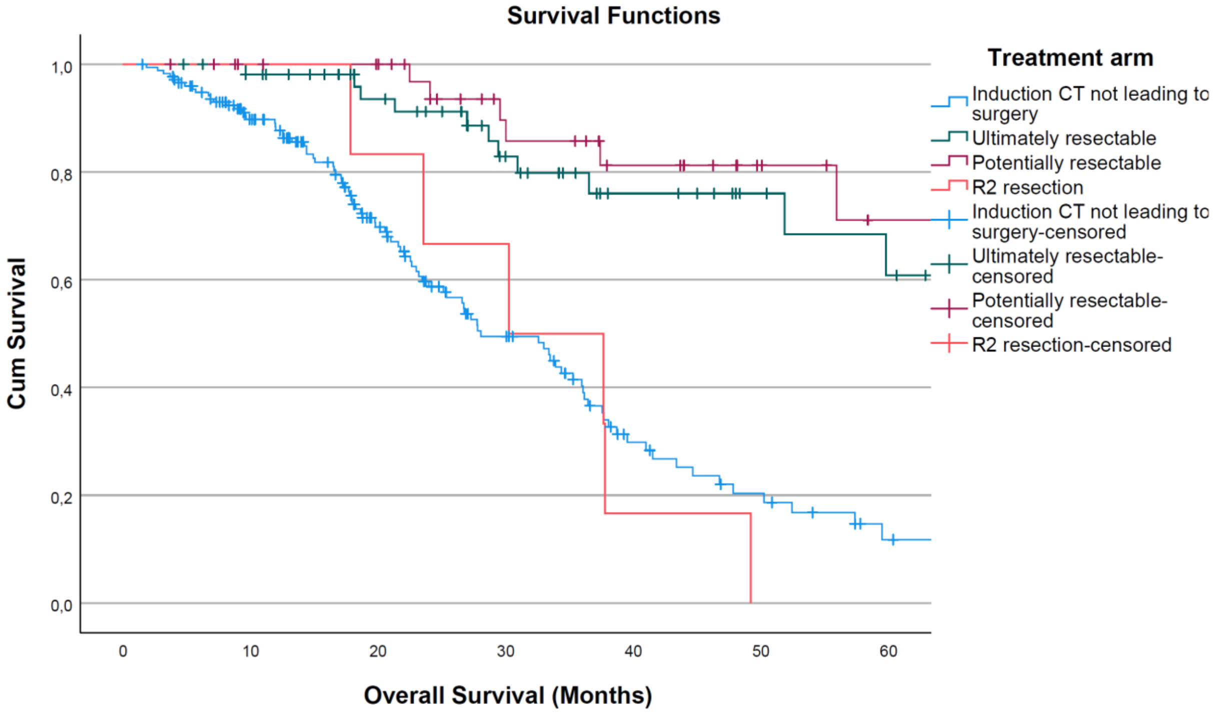

Means and Medians for Survival Time

	Mean[a]				Median			
			95% Confidence Interval				95% Confidence Interval	
Treatment arm	Estimate	Std. Error	Lower Bound	Upper Bound	Estimate	Std. Error	Lower Bound	Upper Bound
Induction CT not leading to surgery	33,078	1,918	29,318	36,838	28,039	3,007	22,145	33,933
Ultimately resectable	62,974	4,336	54,476	71,472	79,379	,000	.	.
Potentially resectable	83,119	8,212	67,024	99,213	.	.	.	.
R2 resection	32,696	4,597	23,687	41,705	30,229	8,645	13,284	47,173
Overall	47,395	3,432	40,668	54,123	37,778	2,535	32,810	42,746

a. Estimation is limited to the largest survival time if it is censored.

Pairwise Comparisons

		Induction CT not leading to surgery		Ultimately resectable		Potentially resectable		R2 resection	
	Treatment arm	Chi-Square	Sig.	Chi-Square	Sig.	Chi-Square	Sig.	Chi-Square	Sig.
Log Rank (Mantel-Cox)	Induction CT not leading to surgery			29,582	<,001	29,187	<,001	,096	,756
	Ultimately resectable	29,582	<,001			,588	,443	12,633	<,001
	Potentially resectable	29,187	<,001	,588	,443			16,457	<,001
	R2 resection	,096	,756	12,633	<,001	16,457	<,001		

4) Figure S4: Kaplan-Meier curves for PFS including all R2 resections

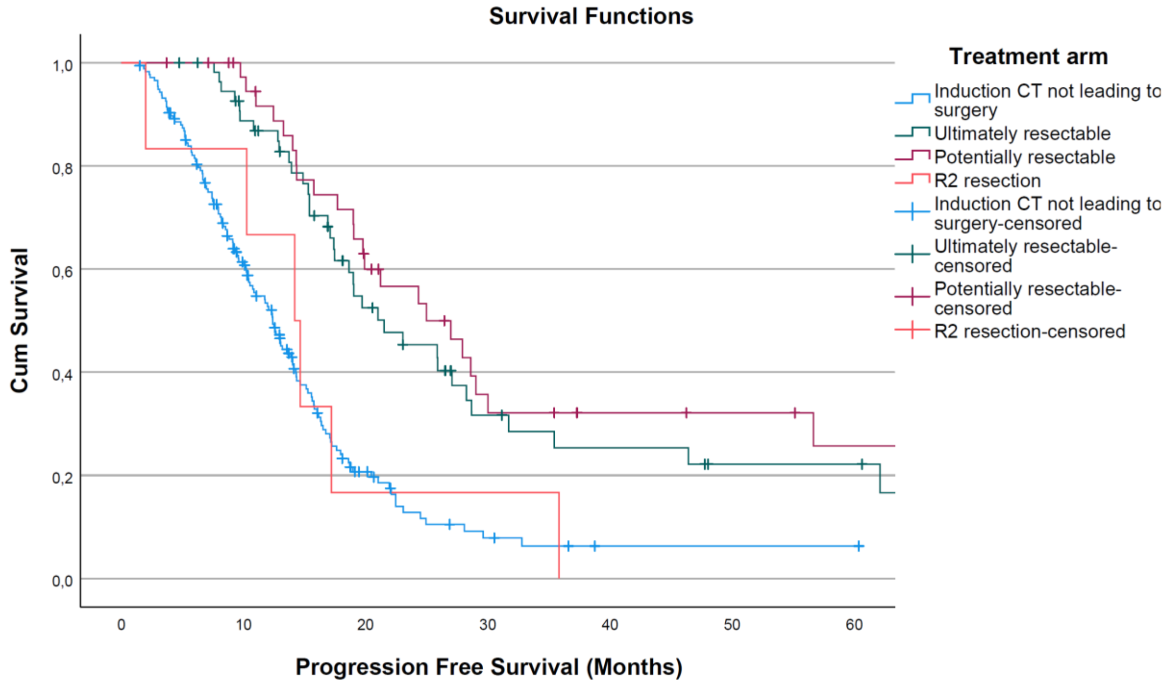

Means and Medians for Survival Time

Treatment arm	Mean[a]				Median			
			95% Confidence Interval				95% Confidence Interval	
	Estimate	Std. Error	Lower Bound	Upper Bound	Estimate	Std. Error	Lower Bound	Upper Bound
Induction CT not leading to surgery	15,483	1,250	13,034	17,932	12,386	,832	10,754	14,017
Ultimately resectable	31,831	3,705	24,570	39,093	21,503	3,548	14,549	28,458
Potentially resectable	42,152	6,813	28,798	55,507	24,967	4,221	16,695	33,240
R2 resection	15,681	4,574	6,715	24,647	14,183	2,682	8,927	19,439
Overall	25,533	2,249	21,124	29,942	15,294	,696	13,929	16,659

a. Estimation is limited to the largest survival time if it is censored.

Pairwise Comparisons

	Treatment arm	Induction CT not leading to surgery		Ultimately resectable		Potentially resectable		R2 resection	
		Chi-Square	Sig.	Chi-Square	Sig.	Chi-Square	Sig.	Chi-Square	Sig.
Log Rank (Mantel-Cox)	Induction CT not leading to surgery			25,718	<,001	25,944	<,001	,045	,832
	Ultimately resectable	25,718	<,001			,645	,422	4,360	,037
	Potentially resectable	25,944	<,001	,645	,422			6,097	,014
	R2 resection	,045	,832	4,360	,037	6,097	,014		

5) **Figure S5: Kaplan-Meier curves for PFS of patients discussed within and outside MDT**

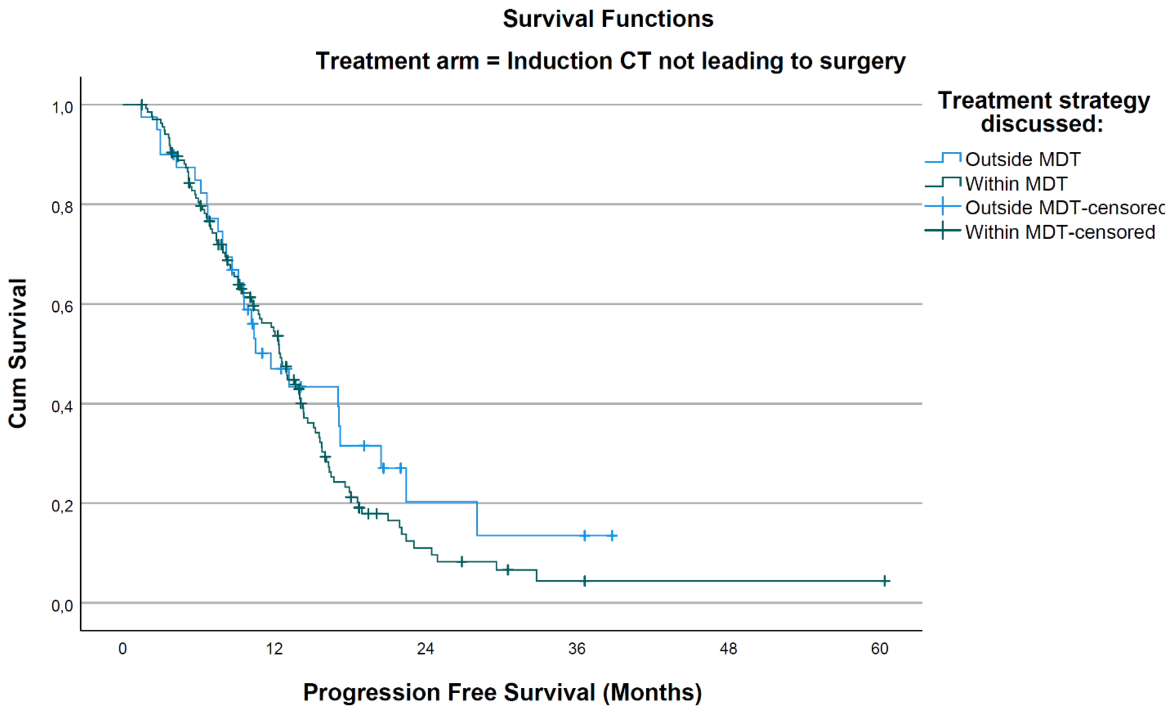

Figure S5A: Kaplan-Meier curves for PFS of patients undergoing induction ST not leading to surgery within and outside MDT

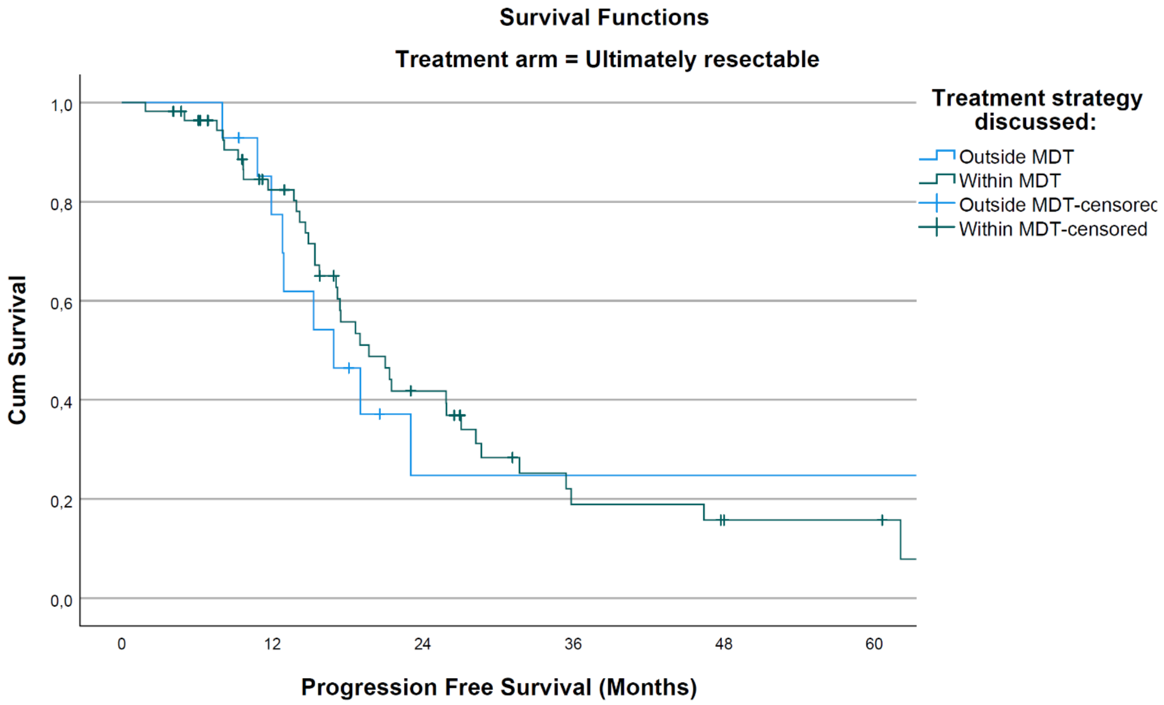

Figure S5B: Kaplan-Meier curves for PFS of ultimately resectable patients within and outside MDT

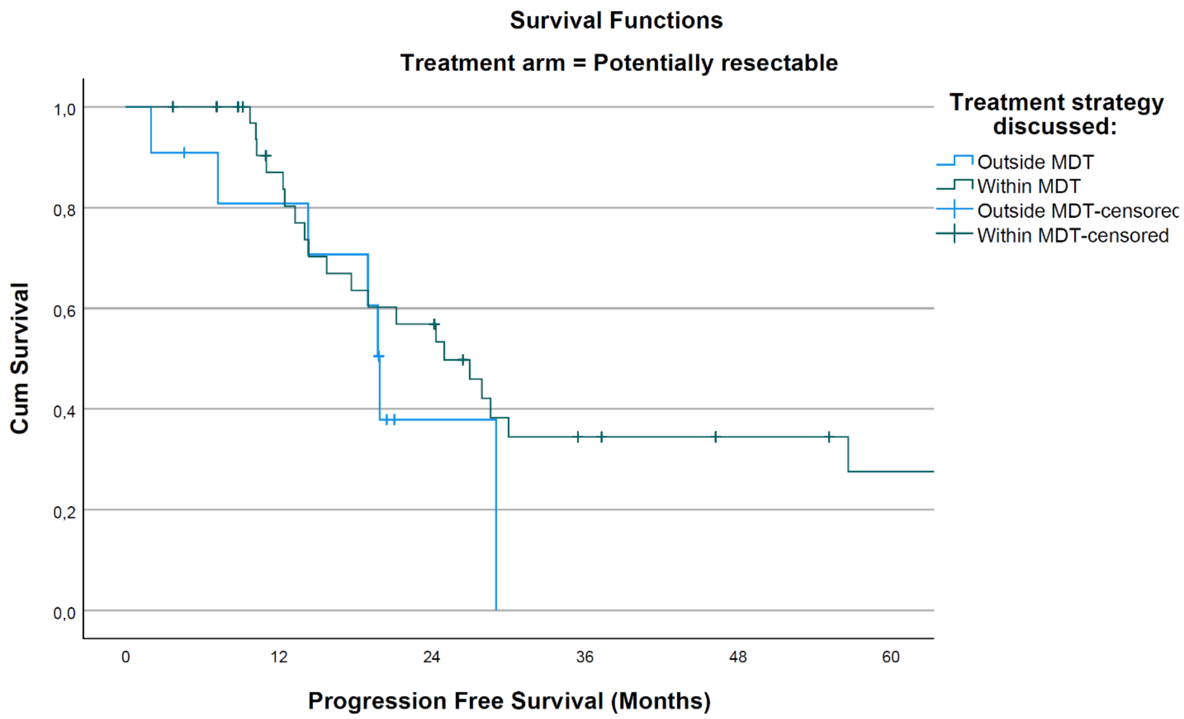

Figure S5C: Kaplan-Meier curves for PFS of potentially resectable patients within and outside MDT

6) **Figure S6: Kaplan-Meier curves for OS of patients receiving different I line ST regimens**

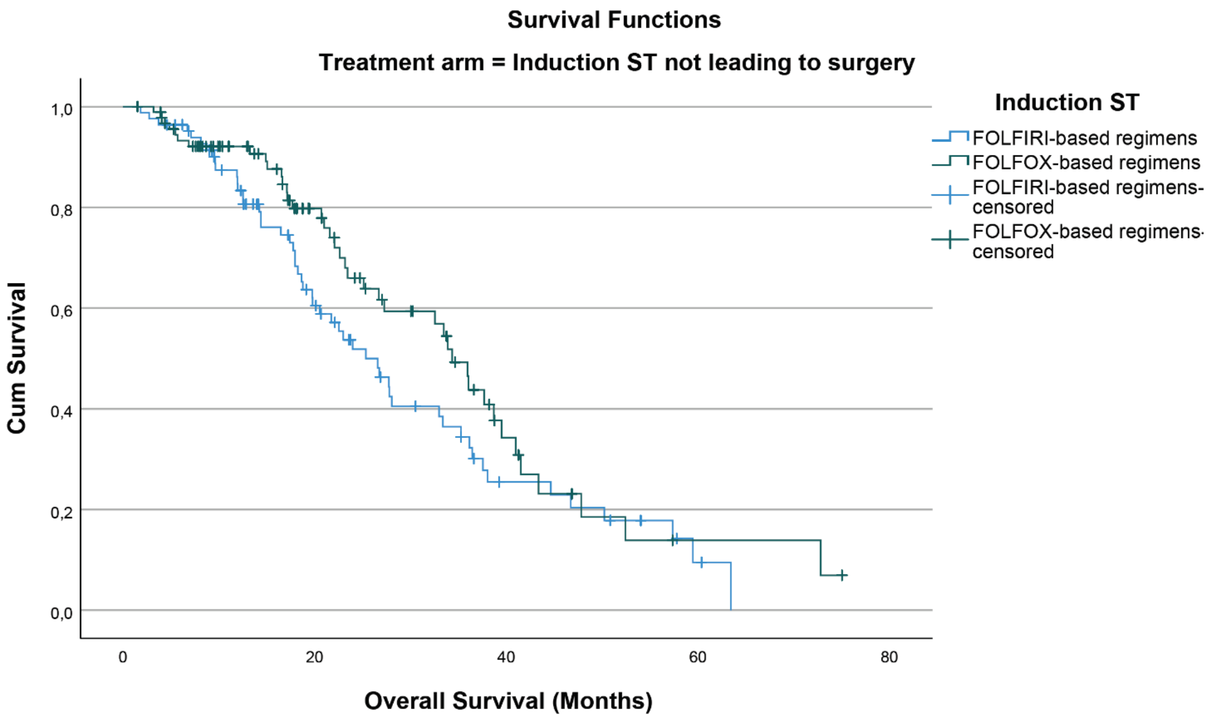

Figure S6A: Kaplan-Meier curves for OS of patients undergoing induction ST not leading to surgery and receiving different I line ST regimens.

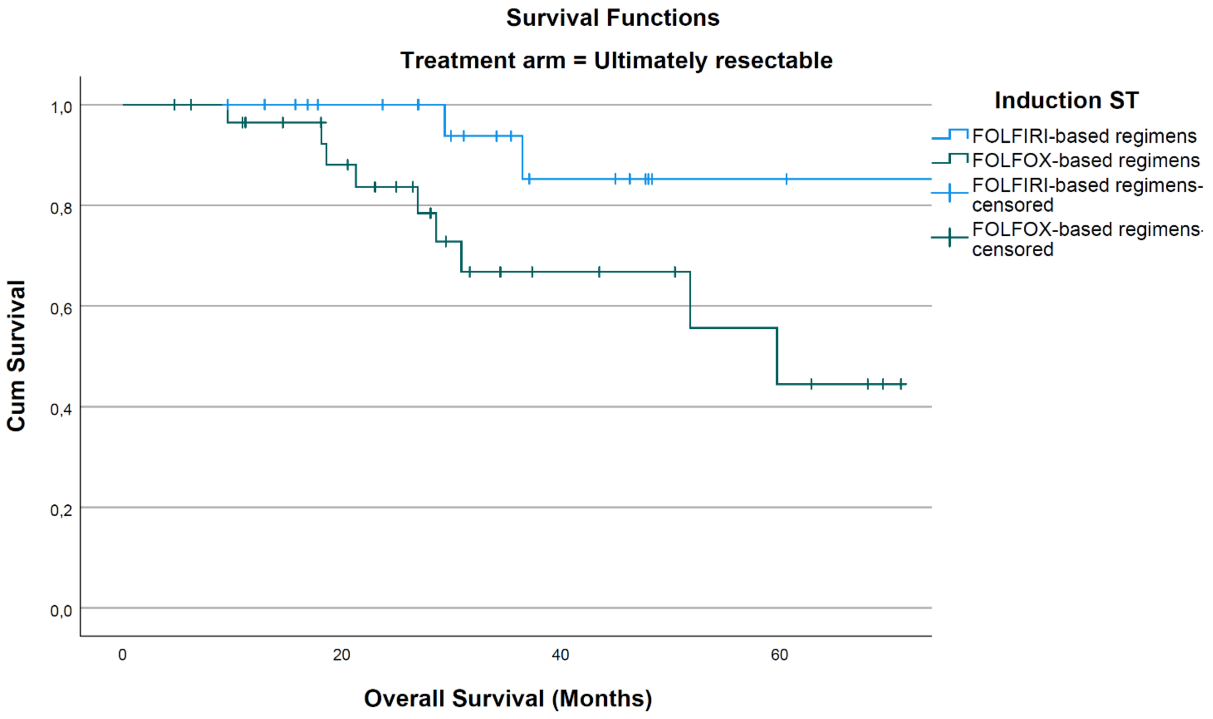

Figure S6B: Kaplan-Meier curves for OS of ultimately resectable patients and receiving different I line ST regimens.

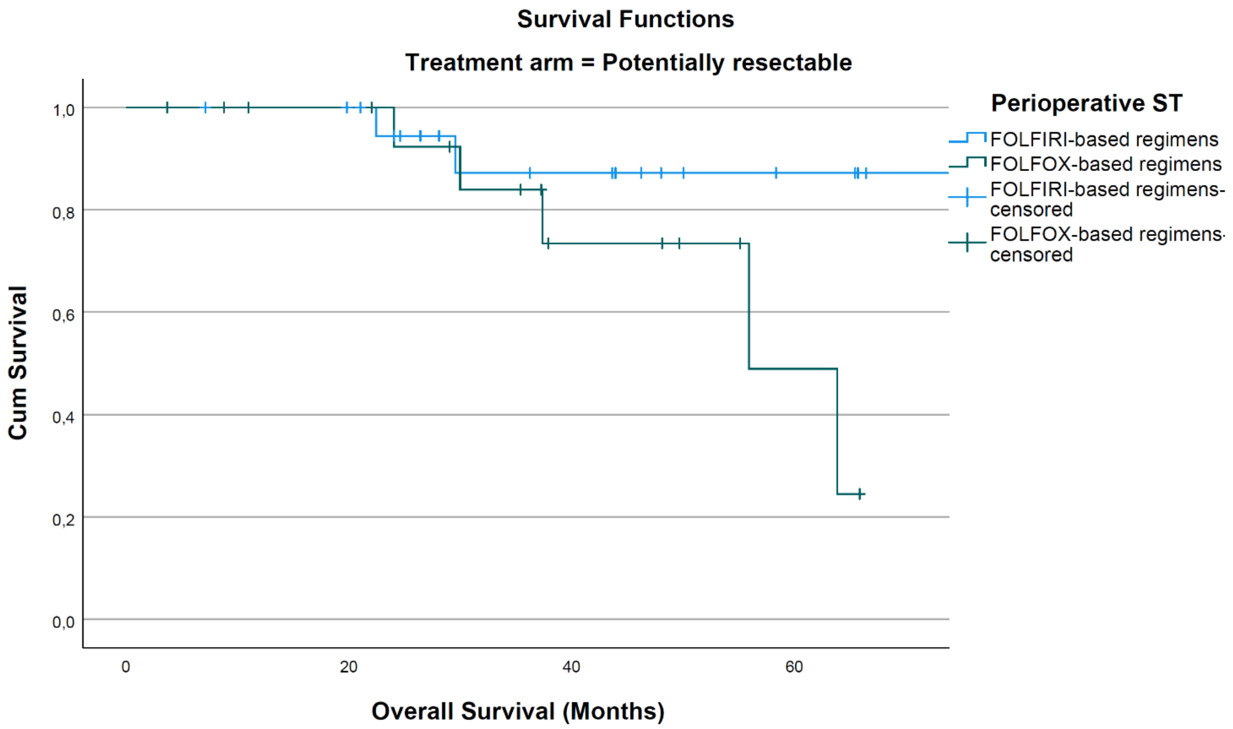

Figure S6C: Kaplan-Meier curves for OS of potentially resectable patients and receiving different perioperative ST regimens.

7) Figure S7: Kaplan-Meier curves for PFS of patients receiving different I line ST regimens

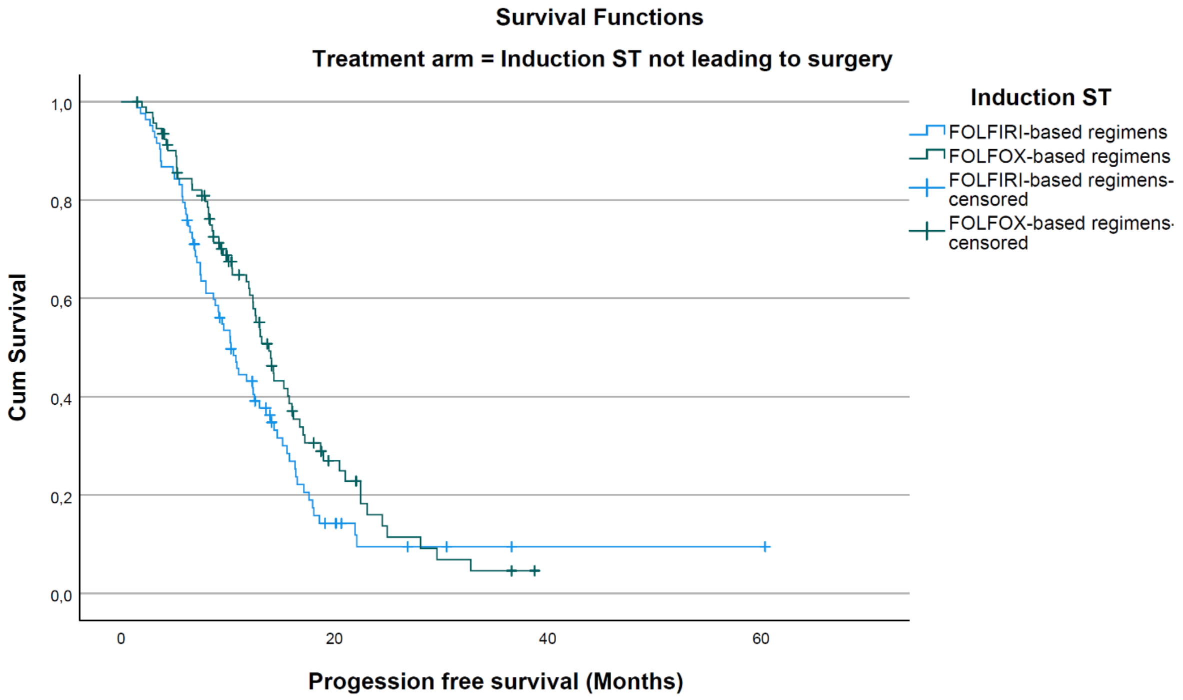

Figure S7A: Kaplan-Meier curves for PFS of patients undergoing induction ST not leading to surgery and receiving different I line ST regimens.

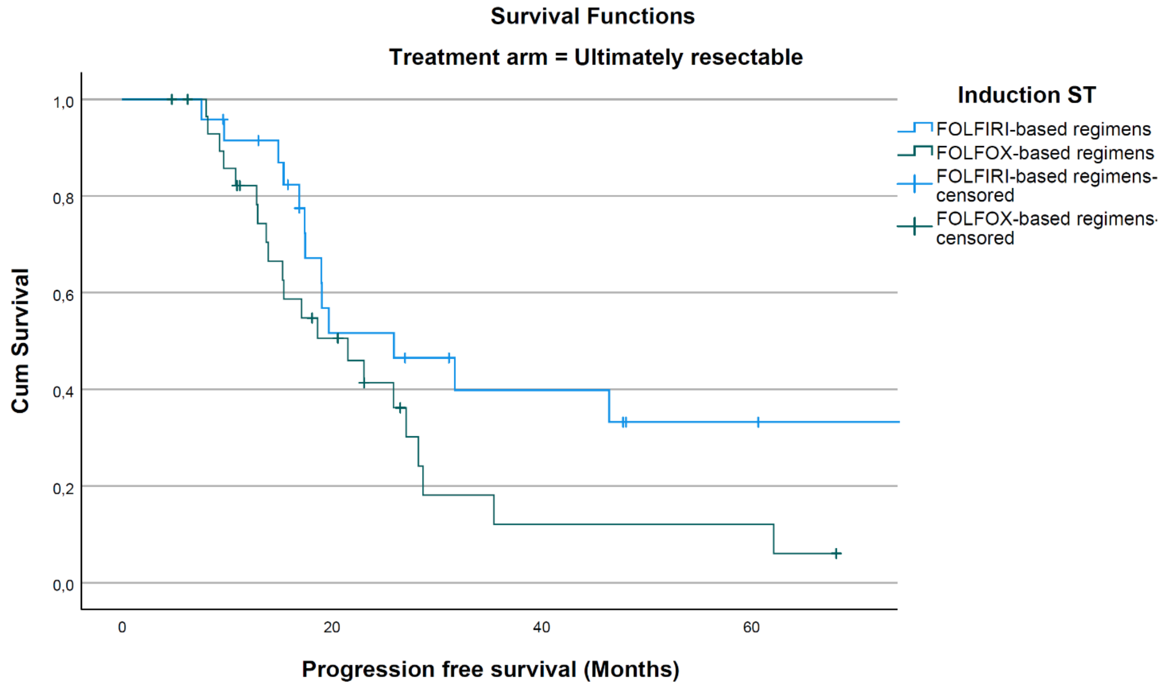

Figure S7B: Kaplan-Meier curves for PFS of ultimately resectable patients and receiving different I line ST regimens.

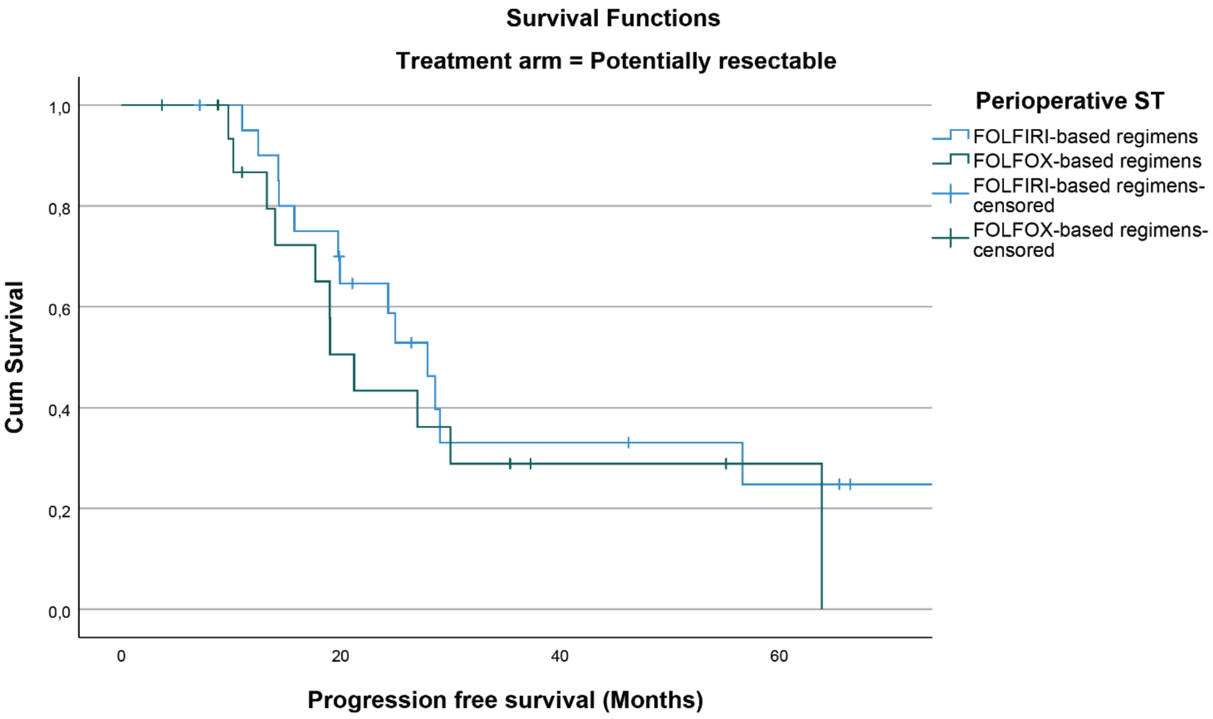

Figure S7C: Kaplan-Meier curves for PFS of potentially resectable patients and receiving different perioperative ST regimens.
